# Influence of Characteristics of Thermoplastic Polyurethane on Graphene-Thermoplastic Polyurethane Composite Film

**DOI:** 10.3390/mi12020129

**Published:** 2021-01-26

**Authors:** Zhi-Min Zhou, Ke Wang, Kai-wen Lin, Yue-Hui Wang, Jing-Ze Li

**Affiliations:** 1 Department of Materials and Food, Zhongshan Institute, University of Electronic Science and Technology of China, Zhongshan 528402, China; zzmzsedu@126.com (Z.-M.Z.); wkzsedu@126.com (K.W.); kevinlin1990@163.com (K.-w.L.); 2 Department of Material and Energy, University of Electronic Science and Technology of China, Chengdu 610054, China; lijingze@uestc.edu.cn

**Keywords:** graphene, thermoplastic polyurethane, near-infrared photothermal response, film

## Abstract

Graphene-thermoplastic polyurethane (G-TPU) composite films were fabricated by traditional blending method and tape casting process with commercial graphene sheets as functional fillers and TPU masterbatches of four different melting points as matrix, respectively. The effects of matrix on the distribution of graphene, the electrical conductivity, and infrared (IR) light thermal properties of the G-TPU composite films were investigated. The experimental results reveal that the characteristics of TPU has little influence on the electrical conductivity of the G-TPU composite films, although the four TPU solutions have different viscosities. However, under the same graphene mass content, the thermal conductivity of four G-TPU composite films with different melting points is significantly different. The four kinds of G-TPU composite films have obvious infrared (IR) thermal effect. There is little difference in the temperatures between the composite films prepared by TPU with melting a point of 100 °C, 120 °C, and 140 °C, respectively; however, when the content of graphene is less than 5 wt%, the temperature of the composite film prepared by TPU with a melting point of 163 °C is obviously lower than that of the other three composite films. The possible reason for this phenomenon is related to the structure of TPU.

## 1. Introduction

In recent years, with the development of electronics, communication, and artificial intelligence industry, flexible conductive composites have attracted extensive attention in academia and industry due to its portability, good biological compatibility, and stretchability and are widely used in sensors, flexible displays, energy devices, medical electronics and integrated circuit, and so on [1,2,3,4,5]. At present, flexible conductive composite materials are usually composed of polymer materials and conductive nanomaterials [4,5,6,7,8]. Polymer materials, such as epoxy, polyimide, and polyurethane, as flexible substrates are mostly insulators and do not have electrical conductivity. Conductive nanomaterials generally include graphene and its derivatives [6,7,8,9], carbon nanotubes [10], metal nanowires [11] and their derivatives [12]. Adding conductive nanomaterials into the matrix or surface of polymer materials can not only solve the shortcomings of the polymer itself, such as brittleness, poor electrical conductivity and thermal conductivity, but also use the special properties of nanomaterials to broaden the application fields of polymer materials [7,13,14,15,16].

Polyurethane is a kind of block polymer composed of hard and soft segments, which has high industrial value and research value due to its excellent oil resistance, abrasion resistance, stretchability and so on [17]. Thermoplastic polyurethane (TPU), as an important branch of polyurethane, shows potential for use in the preparation of perforated membrane because of its excellent tensility, good elastic resilience, and excellent biocompatibility [18,19,20,21]. However, due to its poor electrical conductivity and thermal conductivity, it is difficult to further expand its application fields [19,22].

Graphene (G), a novel two-dimensional honeycomb carbon nanomaterial, has large specific surface area and excellent mechanical, optical, thermal and electrical properties, and also has excellent energy conversion capabilities, such as photothermal conversion, electrothermal conversion, and magnetothermal conversion, is a kind of promising nanomaterial [15,21,23,24]. Therefore, graphene as functional fillers were widely used in to prepare functional materials. Li et al. reported a novel self-healing electronic material with a three-dimensional graphene structure based on Diels-Alders chemistry [1]. Oh et al. reported that they used graphene nanoplates as reinforcing agents and crosslinking platform to react with the furfuryl functional groups in the polyurethane chain via in situ Diels-Alders reaction and synthesized thermally self-healable graphene-nanoplates-polyurethane (GNP-PU) composites [6]. Wang et al. reported that they prepared thermally and mechanically reinforced graphene-thermoplastic polyurethane composites by solution mixing [9]. Luan et al. reported that they filled graphene and carbon nanotubes into TPU to synthesize composites that can be repaired under microwave radiation [20]. Huang et al. reported that they added few-layers graphene into TPU to prepare functional materials that can be healed by microwave, near-infrared light, and electricity [21]. Chen et al. reported that they prepared stretchable and conductive G-TPU nanocomposite foam materials via water vapor induced phase separation technology [18]. Strankowski et al. reported that they used graphene nanoplates and reduced graphene oxide as fillers to prepare two kind of thermally reinforced and mechanically reinforced TPU composites [25]. Cataldi et al. reported that they prepared self-healing flexible conductive cotton textiles by impregnating cotton fabrics in graphene and thermoplastic polyurethane-based dispersions [26]. Roy et al. reported that they prepared mechanically and thermally enhanced composites by incorporating multi-walled carbon nanotubes and graphene hybrid systems into thermoplastic polyurethane [27].

However, most of the previous studies only focused on the mechanical, thermal, and electrical properties of G-TPU composites, while ignoring the electro-thermal and photothermal conversion capabilities of G-TPU composites [1,6,8,9,24,25,26,28,29]. We have reported that the electrical and thermal and self-healing properties of G-TPU conductive film were closely related to the mass content of graphene in the G-TPU film, while the infrared light thermal response performance of G-TPU film has nothing to do with the mass content of graphene in the G-TPU film [25]. In this paper, we fabricated the G-TPU composite films via traditional blending method and tape casting process, then we systematically investigated the effects of the characteristics of TPU on the near-infrared photo-thermal response of G-TPU composite film.

## 2. Materials and Methods

### 2.1. Materials

Graphenesheets (G > 98 wt%) with less than 3 layers and an average particle size less than 10 μm were purchased from Chengdu Jiacai Technology Co., Ltd., Chengdu, China. *N*,*N*-Dimethylformamide (DMF) was purchased from Guangzhou Chemical Reagent Co., Ltd., Guangzhou, China; thermoplastic polyurethane (TPU) masterbatches model number: HF-3H85A-3 (TPU-A), HM85A (TPU-B), and E685C4 (TPU-C), respectively, were purchased from BASF (China) Company Ltd. Guangzhou Branch, Guangzhou, China; ALR CL87A (TPU-D) masterbatches were purchased from Lubrizol Estane Chemical Co. Ltd., Estane Lubrizol, Cleveland, OH, USA.

### 2.2. Methods

#### 2.2.1. Preparation of G-TPU Composite Film

**Figure 1:**
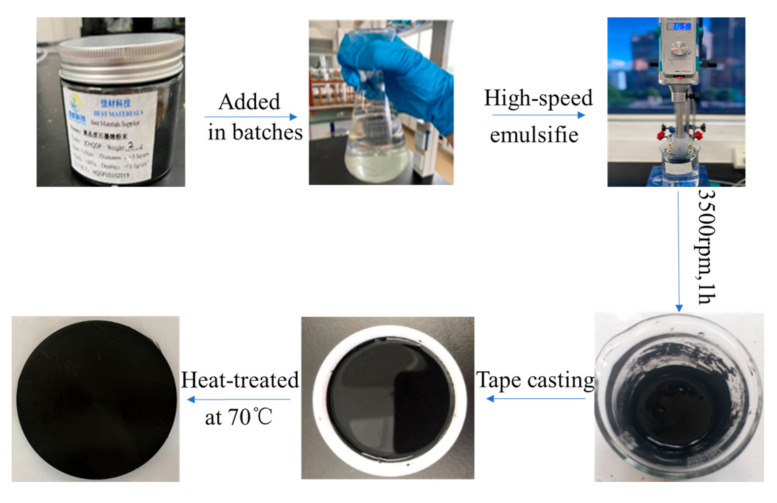
Schematic diagram of the fabrication process of G-TPU composite film.

Typical preparation processes are described as follows: 16 g TPU masterbatches were added into 80 mL DMF, followed by ultrasound and stirring with a stirring rod until completely dissolved. The 0.6 g graphene sheets were added into a flask containing DMF solution under vigorous stirring and ultrasound for 30 min. Then, the TPU solution was added into the above graphene DMF under vigorous agitation and the G-TPU solution was dispersed at 3500 rpm/min for 60 min by a high-speed shear disperser. Finally, the G-TPU solution was poured into the Teflon mold and dried at 70 °C until the weight did not change any more, then the G-TPU composite film were obtained. The G-TPU composite film was peeled off for further testing. G-TPU compound films with different properties were obtained by changing the type of TPU and the mass content of GP. Figure 1 shows a schematic diagram of the fabrication process of G-TPU composite film.

#### 2.2.2. Characterization

Differential scanning calorimetry (DSC) analysis was conducted via simultaneous differential thermal analysis (TA Q2000 V24.1 Build 124, NETZSCH-Gertebau GmbH, Selb, Germany). The weight of sample was 13–15 mg, Gas1: Nitrogen 50.0 mL∙min^−1^, the heating rate was 20 °C/min, and the sample test was heated from −80 °C to 200 °C, then naturally cooled to −80 °C, and then heated to 200 °C. The curves in the figure showed the temperature rise from −80 °C to 200 °C after removing the thermal stress. Scanning electron microscope (SEM, Zeiss sigma 500, Carl Zeiss, Germany), and optical microscope (Nikon LV100, Nikon Co., Ltd., Tokyo, Japan) with a digital camera were used to investigate the microstructure of G-TPU flexible conductive film. The resistance was measured by a four-point probe system (ST2253, Suzhou Jingge Electronics Co., Ltd. Suzhou, China). The resistances of each sample were each measured at twenty different sites and calculated from the average value of those measurements. An infrared thermal imager (UTI384M, range: −20–150 °C, accuracy: ±2 °C, UNI-T China Co., Ltd., Shenzhen, Guangdong, China) was used. The thermal conductivity of sample was measured by a DRL-III heat flow meter instrument (Xiangtan Xiangyi Instrument Co. Ltd., Xiangtan, China) according to the standard ASTM D5470.

## 3. Results and Discussion

### 3.1. Electrical Property of G-TPU Flexible Conductive Film

TPU is a copolymer consisting of both hard and soft segments. Its properties are mainly determined by the monomers, such as the type and crystallinity and copolymer morphologies of hard and soft segments [19,20,21]. The hard section has a direct effect on the mechanical properties of TPU, such as tensile strength, hardness, and modulus; the soft segment determines the elasticity and low temperature resistance of the TPU. Previous studies have shown that graphene can not only strengthen and toughen TPU, but also improve the properties of TPU, such as wear resistance, scratch resistance, heat resistance, aging resistance, electromagnetic shielding property, etc. [6,9,20,21,22,23,24,25,26,27]. The distribution of graphene sheets in TPU and their binding state affect the curing behavior of TPU, which affects electrical and thermal properties and microstructures of the composite films [20,21,22,23]. According to our previous research, the electrical and thermal and infrared light properties of G-TPU composite films are related not only to the mass content of graphene, but also to the initial TPU concentration [25,26]. The initial TPU concentration of 20 wt% is beneficial to obtain the G-TPU composite film with good electrical and thermal properties [25,26]. Here, TPU with the initial concentration of 20 wt% is used to combine graphene with different mass contents. TPU with four different melting points (110 °C, 120 °C, 140 °C, and 163 °C, respectively) were used to study the influence of the characteristics of TPU on the electrical and thermal and infrared light properties of G-TPU composite films.

**Figure 2:**
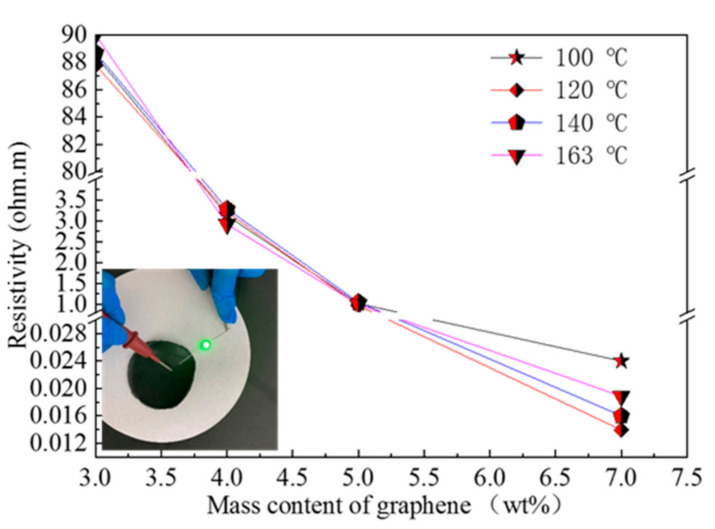
Relationship between the resistivity of the conductive films composed of four different melting points of TPU and the graphene mass content, respectively. The insert is photo of light emitting diode device on the G-TPU conductive film.

Figure 2 shows the relationship between the resistivity of the conductive films composed of four different melting points of TPU and the graphene mass content, respectively. The inset in Figure 2 is photo of light emitting diode device on the G-TPU conductive film. The G-TPU composite films composed of four different melting points of TPU, respectively, were not conductive when the graphene mass content is less than 3 wt%. When the graphene mass content reached 3 wt%, the composite films began to be conductive, but it is clear that the conductivities of composite films have little to do with the characteristics of TPU. We believe that the slight difference in resistivity between different samples is related to the measurement error. When the graphene mass contents reached 3 wt%, 4 wt%, 5 wt%, and 7 wt%, the resistivity of G-TPU flexible conductive films was about 90.0 Ω∙m, 3.0 Ω∙m, 1.1 Ω∙m, and 0.02 Ω∙m, respectively. During heat treatment process of samples, the TPU molecular chains began crosslinking as the DMF volatilized and the mixed slurry gradually became a thin film. With the extension of heat treatment time, the film gradually shrank, making the graphene sheets overlap and stack to form conductive networks. When the mass content of graphene was low (less than 3 wt%), the effective conducting networks could not be formed, so the composite film was non-conductive. When the mass content of graphene reached 3 wt%, a part of the effective conductive pathways were formed, and the flexible conductive film exhibited electrical conductivity, but the resistivity was still high. This indicates that the conductive mechanism of G-TPU composite conductive film conforms to the percolation threshold theory. When the number of graphene sheets overlapping is up to threshold, a considerable ensemble of electrons is finally transported in the entire graphene sheets networks. As the graphene mass content further increases, the number of conductive pathways increases and form more conductive networks, so that the resistivity of G-TPU films gradually decreases until it is stable. The experimental results in Figure 2 show that the distribution of graphene in TPU with different characteristics are not affected by the characteristics of TPU matrix, or it can be said that graphene sheets have similar effects on curing behaviors of TPU. A photo of a light-emitting diode device on the G-TPU film proves good electrical conductivity.

**Figure 3:**
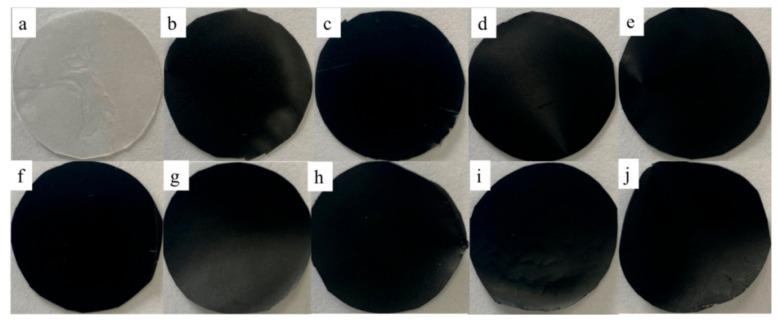
Photos of samples of pure TPU film (melt point of 163 ^o^C, (**a**)), and G-TPU composite films with graphene mass content of 0.1 wt% (**b**), 0.3 wt% (**c**), 0.6 wt% (**d**), 1.0 wt% (**e**), 2.0 wt% (**f**), 3.0 wt% (**g**), 4.0 wt% (**h**), 5.0 wt% (**i**), and 7.0 wt% (**j**), respectively.

Figure 3 shows photos of samples of the pure TPU film (melt point of 163 °C, Figure 3a) and G-TPU composite films with graphene mass content of 0.1 wt% (Figure 3b), 0.3 wt% (Figure 3c), 0.6 wt% (Figure 3d), 1.0 wt% (Figure 3e), 2.0 wt% (Figure 3f), 3.0 wt% (Figure 3g), 4.0 wt% (Figure 3h), 5.0 wt% (Figure 3i), and 7.0 wt% (Figure 3j), respectively. When the graphene mass content is less than 0.6 wt%, the composite film is translucent, after that the composite films become opaque black.

**Figure 4:**
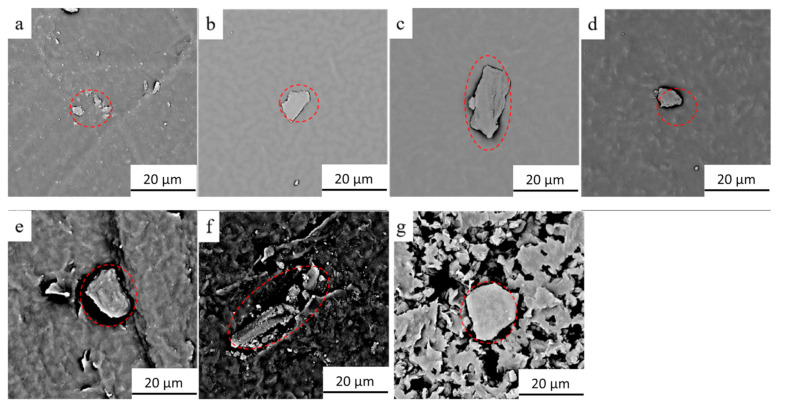
SEM images of surface morphology of the composite films prepared with the initial TPU concentration of 20 wt% and the graphene mass content of 0.3 wt% (**a**), 0.6 wt% (**b**), 2.0 wt% (**c**), 3.0 wt% (**d**), 4.0 wt% (**e**), 5.0 wt% (**f**), and 7.0 wt% (**g**), respectively.

In order to understand the distribution of graphene in TPU, the samples were conducted by SEM. Figure 4 shows SEM images of surface morphology of the composite films prepared with TPU (melt point of 163 °C) and the graphene mass content of of 0.3 wt% (Figure 4a), 0.6 wt% (Figure 4b), 2.0 wt% (Figure 4c), 3.0 wt% (Figure 4d), 4.0 wt% (Figure 4e), 5.0 wt% (Figure 4f), and 7.0 wt% (Figure 4g), respectively. It can be seen from Figure 4 that when the graphene mass content is less than 3 wt%, the TPU strips and individual graphene blocks (red dotted area) are found on the surface of the G-TPU film. When the graphene mass contents reach 3 wt% and 4 wt%, the surfaces of the G-TPU films are obviously roughened and have many particle-like agglomerates, while the surfaces look dense. When the graphene mass contents reach 5 wt% and 7 wt%, more particle-like aggregates are observed and there are many holes between the aggregates, indicating that, due to the insufficient amount of TPU, the gaps between the aggregates are not filled well. Obviously, as the graphene mass content increases, the more graphene sheets overlap with each other, and the more conductive pathways and networks are formed. However, the excessive graphene mass content leads to severe agglomeration of graphene sheets, which leads to the existence of many holes in the composite film.

**Figure 5:**
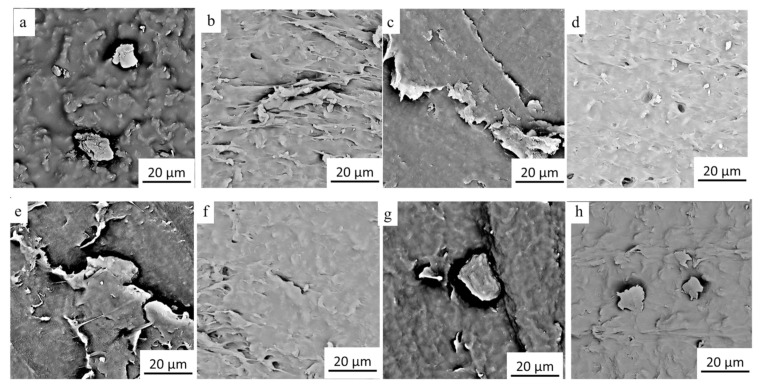
SEM images of top section (above) and cross-section (below) morphologies of the G-TPU films prepared from TPU with melting points of 100 °C (**a**,**b**), 120 °C (**c**,**d**), 140 °C (**e**,**f**), 163 °C (**g**,**h**), respectively, and the graphene mass content of 4 wt%.

Figure 5 shows SEM images of the top section (above) and cross-section (below) morphologies of the G-TPU films prepared from TPU with melting points of 100 °C (Figure 5a,b), 120 °C (Figure 5c,d), 140 °C (Figure 5e,f), 163 °C (Figure 5g,h), respectively, and the graphene mass content of 4 wt%. The graphene sheets overlapped in the TPU matrix can been observed and there is no significant difference in the distribution of graphene sheets among the four kinds of TPU.

### 3.2. Thermal Property of G-TPU Composite Film

**Figure 6:**
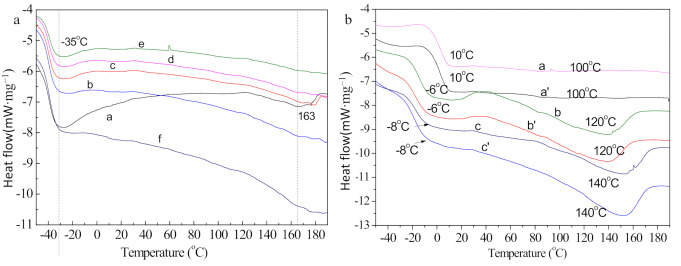
DSC measurements of G-TPU composite films prepared from TPU with melting points of 163 °C and the graphene with different mass contents (Figure 6a) and G-TPU composite films prepared from TPU with the melting point of 100 °C (Figure 6b, curve a, a’), 120 °C (Figure 6b, curve b, b’), 140 °C (Figure 6b, curve c, c’), respectively, and the graphene mass content of 4 wt% (Figure 6b).

Figure 6 shows the DSC measurements of G-TPU composite films prepared from TPU with melting points of 163 °C and the graphene with different mass contents (Figure 6a) and G-TPU composite films prepared from TPU with the melting point of 100 °C (Figure 6b, curve a, a’), 120 °C (Figure 6b, curve b, b’), and 140 °C (Figure 6b, curve c, c’), respectively, and the graphene mass content of 4 wt% (Figure 6b). Seen from curve a in Figure 6a, two melting peaks of about −35 °C and 163 °C were observed in the curve a, which are related to glass-transition temperature (T_g_) and the melt (T_m_) that occurs in the soft segment and hard segment micro-crystalline area, respectively. With the increase of the graphene in the G-TPU, both the glass-transition temperature and the melting point of TPU are almost constant, but the ∆H_m_ increases, indicating that the graphene affects the curing behavior of TPU and facilitates the molecular chain movement. However, the degree of influence on the molecular chain movement is small, not enough to make the yield point temperature change when the polyurethane material is heated. As seen from Figure 6b, comparing to the pure TPU films with different melting points, the glass-transition temperature and the melting point of the G-TPU composite films composed of the TPU with different melting points do not have an obvious difference. The above experimental results indicate that the graphene has a certain effect on the curing behavior of polyurethane, i.e., the motion of soft and hard segments, but does not affect the properties of soft and hard segments.

**Figure 7:**
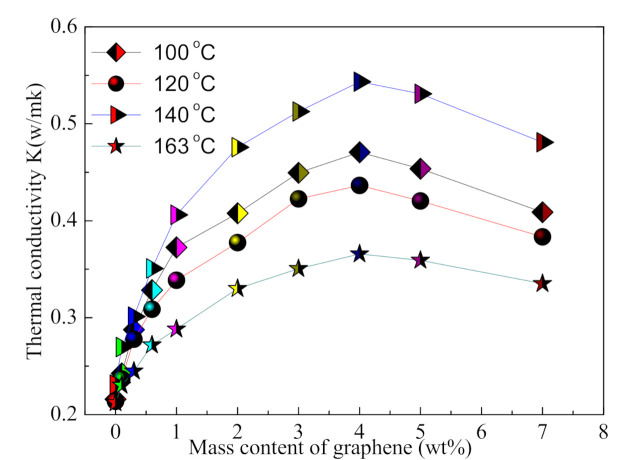
Relationship between the mass content of graphene in the G-TPU film and the thermal conductivities of the pure TPU and the G-TPU composite films with the melting points of TPU of 100 °C, 120 °C, 140 °C, and 163 °C, respectively.

Figure 7 shows the relationship between the mass content of graphene in the G-TPU film and the thermal conductivities of the pure TPU and the G-TPU composite films with the melting point TPU of being 100 °C, 120 °C, 140 °C, and 163 °C, respectively. The thermal conductivities of the pure TPU with the melting point of TPU at 100 °C, 120 °C, 140 °C, and 163 °C, respectively, are 0.2137 W·m^−1^·K^−1^, 0.231 W·m^−1^·K^−1^, 0.2123 W·m^−1^·K^−1^, and 0.2158 W·m^−1^·K^−1^, respectively. The thermal conductivities of the G-TPU composite films with the melting point of TPU at 100 °C, 120 °C, 140 °C, and 163 °C, respectively, first increase and then decrease with the increases of the mass content of graphene in G-TPU film and when the graphene mass content in G-TPU film is 4 wt%, and the thermal conductivity of the G-TPU film reaches the maximum, which are 0.4365 W·m^−1^·K^−1^, 0.4706 W·m^−1^·K^−1^, 0.5434 W·m^−1^·K^−1^, and 0.3657 W·m^−1^·K^−1^, increasing by a factor of 2.04, 2.04, 2.56, 1.69, respectively. As mentioned above, when the graphene content in the composite film is lower than 4 wt%, the graphene sheets overlap and stack to form thermal conductive pathways. As the graphene mass content in the G-TPU film increases to 4 wt%, the graphene sheets show an obvious agglomeration phenomenon, and then a large number of thermal conductive pathways are destroyed [26], and the thermal conductivity of the G-TPU film decreases.

It is worth noting that the thermal conductivity of the G-TPU films prepared from the TPU with different melting points was significantly different from the variation of their conductivity (as shown in Figure 2). The thermal conductivities of the G-TPU films are as follows: K_G-TPU (120 °C)_ > K_G-TPU (100 °C)_ > K _G-TPU (140 °C)_ > K_G-TPU (163 °C)_.

### 3.3. Infrared Light Thermal Response Performances of G-TPU Films

**Figure 8:**
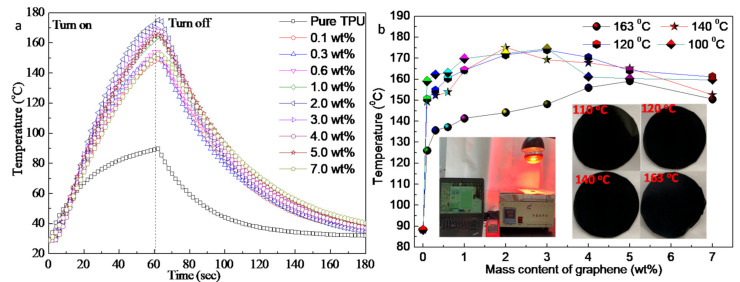
(**a**) Relationship of the temperature of the G-TPU composite films prepared from melting point TPU of 140 °C and with different mass contents of graphene under the operation of IR lamp, and (**b**) the maximum temperature of the G-TPU composite films prepared from TPU with different melting points and the mass content of graphene. The insets are photos of the tested sample and samples at maximum temperature.

Further, we studied the infrared (IR) light thermal response performances of G-TPU films prepared from TPU with different melting points. For the IR light thermal response experiment, we irradiated the samples with an IR lamp (light intensity of 5 × 10^−4^ W∙cm^−2^). Figure 8a shows the relationship of the temperature of the G-TPU composite films prepared by melting point of TPU of 140 °C and with different mass contents of graphene under the operation of IR lamp. The IR lamp was turned on for 60 s and turned off. We also measured the G-TPU composite films prepared from the melting point of TPU of 100 °C, 140 °C, and 163 °C with the different mass contents of graphene, respectively (Figures S1–S3). Figure 8b shows the relationship of maximum temperature of the G-TPU composite film prepared by TPU with different melting points and the mass contents of graphene. The insets in Figure 8b show photos of the tested sample and samples at maximum temperature.

**Figure 9:**
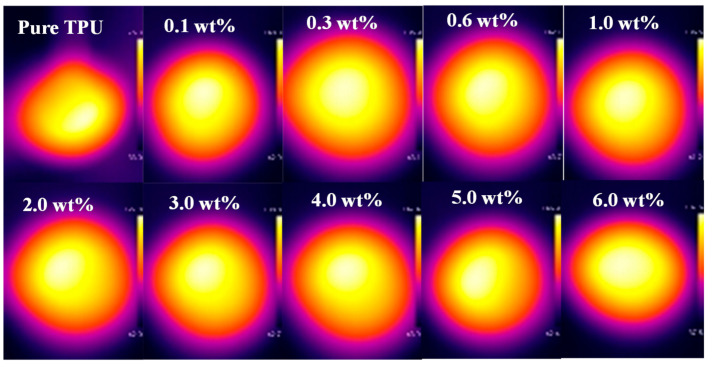
Infrared images of the pure TPU and G-TPU films with different mass contents of graphene sheets.

As seen from Figure 8, same as our previous research results, when graphene sheets were added into TPU, the temperature of the G-TPU composite films increase significantly under IR irradiation, indicating that the composite films have obvious IR irradiation response characteristics. With the increase of graphene mass content, the temperatures of the composite film first increase and then decrease, except that the temperature of the composite film prepared from TPU with a melting point of 163 °C increases with the increase of graphene mass content. The temperature of the pure TPU film heated for 60 s reached 89.6 °C, while, the temperature of G-TPU composite films with the mass content of graphene of 0.1 wt% and 2 wt% reached 161.8 °C and 175.6 °C, increasing by 1.80 times and 1.96 times, respectively. Although the temperatures of the composite films were far beyond the melting point of pure TPU, no melting of the samples is observed (see the photos of samples at maximum temperature in Figure 8b). The above experimental results show that graphene not only promotes the IR irradiation thermal effect of the composite film, but also significantly improves the heat resistance of TPU. There is little difference in the temperatures between the composite films prepared by TPU with melting a point of 100 °C, 120 °C, and 140 °C, respectively. However, when the mass content of graphene is less than 5 wt%, the temperature of the composite film prepared from TPU with a melting point of 163 °C is obviously lower than that of the other three composite films. The possible reason for this phenomenon is related to the structure characteristics of TPU. The morphologies of TPU include hard and soft segments. TPU with a melting point of 163 °C has a higher hard segment content, which affects the heat transfer of graphene. Figure 9 displays the IR images of the G-TPU films heated for 60 s by IR irradiation. The IR images show that the distribution of heat conduction channels formed by graphene sheets is uniform compared with the microstructures of G-TPU films in Figure 4 and Figure 5. It can be seen that a certain amount of graphene sheets agglomeration has little effect on the distribution of the electrical conductivity and heat conduction channels formed by graphene sheets.

## 4. Conclusions

Graphene-thermoplastic polyurethane (G-TPU) composite films were fabricated by traditional blending method and tape casting process with commercial graphene sheets as functional fillers and TPU masterbatches of four different melting points as matrix, respectively. The experimental results show that the characteristics of TPU has little influence on the electrical conductivity of the G-TPU composite films prepared with TPU masterbatches of four different melting points, respectively. However, the thermal conductivity of four G-TPU composite films with different melting points is significantly different in the condition of the same mass content of graphene. When the graphene mass content in G-TPU film is 4 wt%, the thermal conductivities of the four G-TPU films reach the maximum, which are 0.4365 W∙m^−1^∙K^−1^, 0.4706 W∙m^−1^∙K^−1^, 0.5434 W∙m^−1^∙K^−1^, and 0.3657 W∙m^−1^∙K^−1^, increasing by a factor of 2.04, 2.04, 2.56, 1.69, respectively. The four G-TPU composite films have obvious infrared (IR) thermal effect. The temperature of G-TPU composite films with the mass content of graphene of 0.1 wt% and 2 wt% reach 161.8 °C and 175.6 °C, increasing by 1.80 times and 1.96 times, respectively. There is little difference in the temperatures between the composite films prepared by TPU with melting a point of 100 °C, 120 °C, and 140 °C, respectively; however, when the content of graphene is less than 5 wt%, the temperature of the composite film prepared by TPU with a melting point of 163 °C is obviously lower than that of the other three composite films. The possible reason for this phenomenon is related to the structure of TPU.

## Supplementary Materials

The following are available online at https://www.mdpi.com/2072-666X/12/2/129/s1, Figure S1: Relationship of the temperature of the G-TPU composite films prepared by melting point TPU of 100 °C and different mass contents of graphene under the operation of IR lamp; Figure S2: Relationship of the temperature of the G-TPU composite films prepared by melting point TPU of 100 °C and different mass contents of graphene under the operation of IR lamp; Figure S3: Relationship of the temperature of the G-TPU composite films prepared by melting point TPU of 163 °C and different mass contents of graphene under the operation of IR lamp.
