# Supplementary material for: Influence of Characteristics of Thermoplastic Polyurethane on Graphene-Thermoplastic Polyurethane Composite Film
Source: Micromachines (Basel). 2021 Jan 26;12(2):129. doi: 10.3390/mi12020129 (PMC7911175; doi:10.3390/mi12020129)
Supplement: Supplementary file 1 [file micromachines-12-00129-s001.pdf]

micromachines

Supplementary Materials

Influence of Characteristics of Thermoplastic Polyurethane on Graphene-Thermoplastic Polyurethane Composite Film

Zhi-Min Zhou [1,2], Ke Wang [1], Kai-wen Lin [1], Yue-Hui Wang [1,*] and Jing-Ze Li [2]

[1] Department of Materials and Food, Zhongshan Institute, University of Electronic Science and Technology of China, Zhongshan 528402, Guangdong, China; zzmzsedu@126.com (Z.-M.Z.); wkzsedu@126.com (K.W.); kevinlin1990@163.com (K.-w.L.)
[2] Department of Material and Energy, University of Electronic Science and Technology of China, Chengdu 610054, China; lijingze@uestc.edu.cn
* Correspondence: wangzsedu@126.com; Tel.: +86-15-900-020-061

Citation: Zhou, Z.-M.; Wang, K.; Lin, K.-w.; Wang, Y.-H.; Li, J.-Z. Influence of Characteristics of Thermoplastic Polyurethane on Graphene-Thermoplastic Polyurethane Composite Film. *Micromachines* **2021**, *9*, 129. https://doi.org/10.3390/mi12020129

Received: 20 December 2020
Accepted: 24 January 2021
Published: 26 January 2021

Publisher's Note: MDPI stays neutral with regard to jurisdictional claims in published maps and institutional affiliations.

Copyright: © 2021 by the authors. Licensee MDPI, Basel, Switzerland. This article is an open access article distributed under the terms and conditions of the Creative Commons Attribution (CC BY) license (http://creativecommons.org/licenses/by/4.0/).

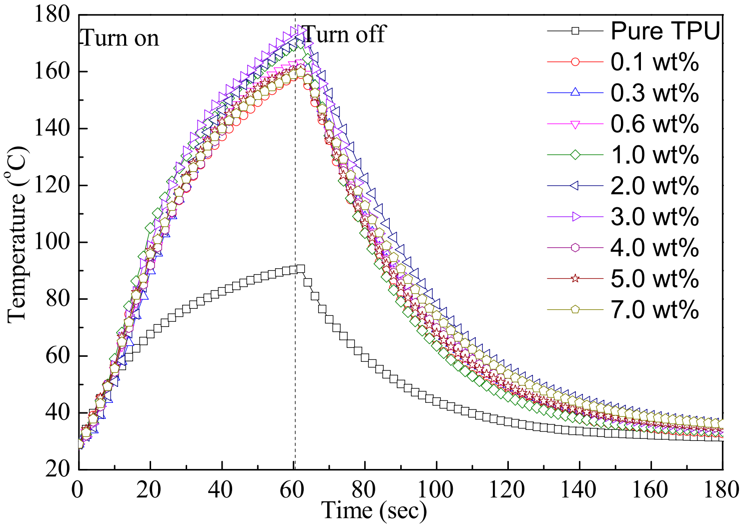

Figure S1. S Relationship of the temperature of the G-TPU composite films prepared by melting point TPU of 100 °C and different mass contents of graphene under the operation of IR lamp.

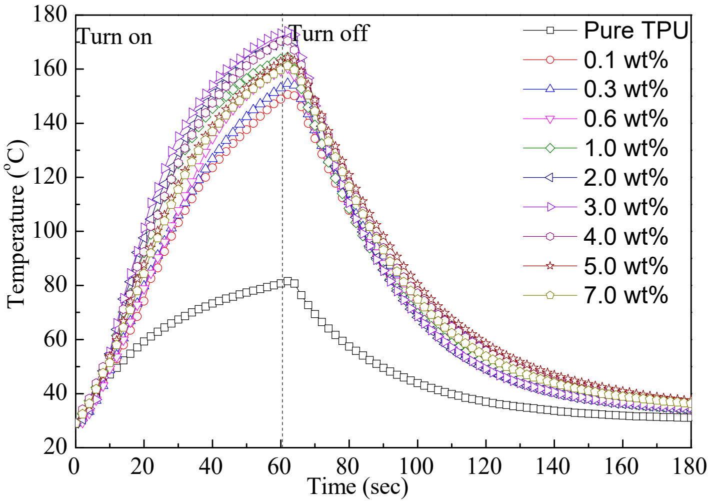

Figure S2. S Relationship of the temperature of the G-TPU composite films prepared by melting point TPU of 100 °C and different mass contents of graphene under the operation of IR lamp.

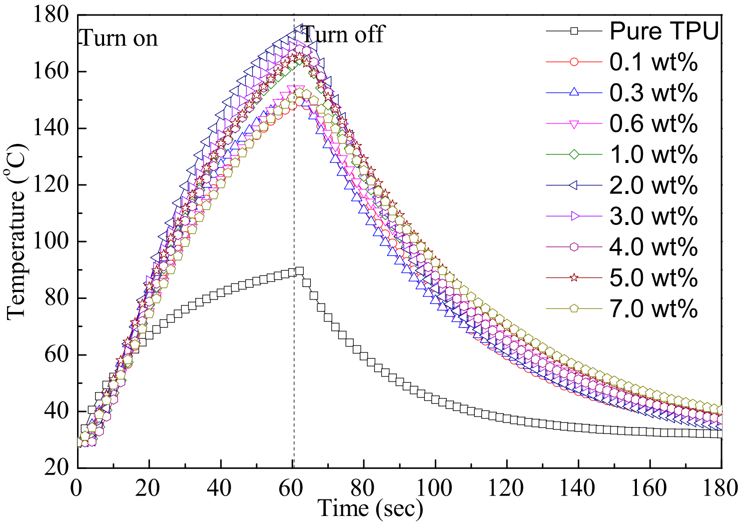

Figure S3. S Relationship of the temperature of the G-TPU composite films prepared by melting point TPU of 163 °C and different mass contents of graphene under the operation of IR lamp.
